# To save the saver: facilitating school counselors’ occupational well-being through multi-dimensional support and basic psychological needs

**DOI:** 10.3389/fpsyg.2024.1475931

**Published:** 2024-10-11

**Authors:** Lina Feng, Yi Liu, Haoyan Huang

**Affiliations:** ^1^Moral Education Research Center, Beijing Institute of Educational Sciences, Beijing, China; ^2^Faculty of Education, Beijing Normal University, Beijing, China; ^3^Faculty of Educational Science, University of Helsinki, Helsinki, Finland

**Keywords:** school counselors, occupational well-being, situational support, inter-individual support, basic psychological needs

## Abstract

School counselors play an increasingly crucial role in protecting students’ mental health. However, many of them have experienced poor well-being in their occupations, which undermines their efficacy in mental protection. To identify the most significant well-being facilitators, this study examined associations between school counselors’ occupational well-being and situational, inter-individual and intra-individual supports, as well as further explored their dynamics. A total of 1,443 Chinese school counselors (66.6% part-time) from a regionally representative dataset were selected, and their work engagement, pressure and satisfaction were surveyed as well-being indicators. Dominance analysis revealed that (1) organizational support and occupational empowerment were consistently robust facilitators for most psychological needs and well-being indicators, (2) students were the most critical supporters for occupational well-being, particularly for part-time counselors; and (3) autonomy and competence were more central needs than relatedness in transferring supports to occupational well-being. Findings implied the most effective strategies to benefit school counselors’ occupational well-being, both in general and across different work cohorts.

## 1 Introduction

Children and adolescents face increasing mental health risks in contemporary society. Recent survey revealed that over 25% of school-age students have suffered from depression or other mental health problems (Racine et al., 2021). To tackle this increasingly serious risk, school counselors have been widely employed in the educational system and have become increasingly crucial, and play vital roles in students’ mental health care (Dimmitt and Wilkerson, 2012; Kang-Yi et al., 2018). Nonetheless, the working efficacy of school counselors has been dramatically undermined by insufficient occupational well-being. The substantial time spent addressing students’ mental health issues may unconsciously exacerbate school counselors’ occupational well-being. The increasing demand for their services and the lack of supporting resources further exacerbate this mental risk (Watson, 2024); consequently, studies have indicated that numerous school counselors have suffered from mental health problems in their occupation (Tay et al., 2018). As such, how to effectively support school counselors’ occupational well-being has become a crucial research topic that remains under-examined.

To fulfill the research gaps, this study aimed to identify the prominent facilitators of school counselors’ occupational well-being by examining the contributions of situational, inter-individual, and intra-individual (i.e., focusing on basic psychological needs) supports, based on the Demand-resource Model. It further explored the mediating role of three basic psychological needs from Self-determination Theory (i.e., autonomy, competence, and relatedness) and investigated the distinctive patterns of these results between full-time and part-time school counselors. A survey was conducted with 1,443 school counselors from a regionally representative dataset in Beijing, China, as they may face more serious mental risks in their occupation than school counselors in developed countries (Yu and Zhang, 2021; Zhang and Gan, 2018).

### 1.1 The contexts of Chinese school mental health service and counselors

Mental health service is increasingly crucial in school education, which aims to monitor students mental health conditions and provide timely intervention (Green et al., 2013; Sanchez et al., 2018). School mental counselors, the primary service providers in school, play a frontline role in the early identification and prevention of students’ mental issues, thereby protecting their mental well-being (Green et al., 2013; Sanchez et al., 2018). Despite its significance, mental health service has long received insufficient attention in Chinese school education until recent years, with unclear boundaries regarding school mental counselors’ occupational responsibilities and related management (Yu and Zhang, 2021; Zhang and Gan, 2018). For instance, numerous Chinese schools allocate heavy and ambiguous tasks to school counselors (e.g., attributing students’ moral education to their responsibility) and offer few opportunities for their professional development (e.g., with fewer promotion prospects than school teachers; Kang and Wan, 2017). These challenges exacerbate the occupational well-being of Chinese school counselors, further threatening their capabilities to support children’s mental health.

Moreover, due to the shortage of professional counselors, many schools have to require their subject teachers to undertake counseling tasks part-time (Kang and Wan, 2017; Yu and Zhang, 2021; Zhang and Gan, 2018). This phenomenon commonly exists in most regions of China, including the capital [i.e., in this regionally-representative survey, 961 (66.6%) schools in Beijing only have part-time counselors]. As a consequence, these part-time school counselors often bear much heavier workloads and suffer from more severe mental health risks in their occupation, necessitating specific attention in research.

### 1.2 Theoretical framework

To comprehensively understand the factors affecting school counselors’ occupational well-being, this study employed the Demand-resources Model as the basis of theoretical framework. The Demand-resource Model posits two distinct processes that impact occupational well-being. The “demand” relates to an effort-driven energetic process that exhaust one’s energy and lead to stress, burnout and diminished mental health, whereas the “resource” refers to a motivational process where the availability of resources promotes work engagement and satisfaction (Salmela-Aro et al., 2016; Salmela-Aro and Upadyaya, 2014). This model underscores that the balance between demands and resources is a key determinant of occupational well-being, in which sufficient resources or supports can compensate for the detriments due to heavy work demands (Salmela-Aro et al., 2022). Considering the difficulties in reducing working demands for school counselors in Chinese schools, this model implies that providing more abundant resources is a more feasible approach to achieve demand-resource balance, and consequently protect their well-being in occupation.

Moreover, the Demands-resources Model proposes that supporting factors (resources) for well-being are present at multiple levels, including situational (or environmental), inter-individual, and intra-individual levels (Salmela-Aro et al., 2016; Salmela-Aro et al., 2022). Since these supporting factors jointly influence school counselors’ well-being in occupation, the present study comprehensively examined their associations with occupational well-being indicators, to identify the most effective well-being facilitators.

### 1.3 Associations of situational support with school counselors’ occupational well-being

Situational support for school counselors is represented by organizational support, occupational empowerment, and occupational values in this study, as these factors are more malleable in the workplace. Organizational support is defined as a comprehensive supportive environment in which employees’ contributions are valued, and their development and well-being needs are addressed (Eisenberger et al., 1986; Rhoades and Eisenberger, 2002). It can provide work autonomy, development opportunities and appraisal feedback, which are crucial facilitators of occupational well-being (Chen et al., 2022). Perceiving this support is also beneficial to employees’ positive emotional experience, stress resilience and occupational commitment (Nong et al., 2022; Singh et al., 2018). Given these benefits, prior studies have identified organizational support in schools as key driver of staff well-being (Lauermann and König, 2016).

Apart from organizational support, it is also crucial for school counselors to perceive their work as influential and valuable; thus, perceived occupational empowerment and value from the environment are important. Occupational empowerment means that school counselors have the freedom of choice in their occupation, participate in school decision-making, and influence goal achievement (Skår, 2010; Van Bogaert et al., 2016). When perceiving robust occupational empowerment, school counselors tend to feel that their work makes sense and generate stronger feelings of satisfaction and achievement in their occupation (Leggat et al., 2010; Smith et al., 2010). Furthermore, they are more likely to be highly motivated and dedicate themselves to their work with fewer negative emotions (e.g., stress and depression; Papathanasiou et al., 2014; Tajvar et al., 2015).

Similarly, occupational value also benefits school counselors’ well-being by enhancing their occupational motivation and sense of achievement (Fute et al., 2022). Known as the perceived significance and meaningfulness of one’s occupation (Jin and Rounds, 2012), occupational value serves as a key motivational factor in school counselors’ work selection, engagement, and satisfaction (Balsamo et al., 2013; Sortheix et al., 2015; Sortheix et al., 2013). School counselors with high perceived occupational value also generate more positive affect which is beneficial to their occupational satisfaction and well-being (Caricati et al., 2014; Moniarou-Papaconstantinou and Triantafyllou, 2015).

### 1.4 Associations of inter-individual support with school counselors’ occupational well-being

Regarding inter-individual support, the Demands-resources Model indicates that significant others (e.g., leaders, colleagues, students, and family members) play crucial roles in occupational well-being (Salmela-Aro et al., 2016; Salmela-Aro et al., 2022; Song et al., 2023). Leaders usually have more power than other significant others to reduce work demands (e.g., workloads and burdens) and provide abundant resources in schools (e.g., nurturing opportunities, autonomy and, collaborative leadership). Thus, their support directly enhances school counselors’ occupational well-being (Richards et al., 2018; Wong and Zhang, 2014). However, as leader support is typically hierarchical, it can sometimes trigger work demands and pressure, especially when leaders’ expectations overwhelm school counselors’ capabilities (Brackett et al., 2010; Richards et al., 2018; Wang et al., 2016).

In contrast, support from colleagues benefits well-being in a different way due to its more equal and collaborative nature. Specifically, colleague support can help school counselors acquire more occupational experience, better cope with professional tasks, and perceive a stronger sense of relatedness. These benefits, in turn, diminish school counselors’ stress and burnout and improve self-efficacy and satisfaction in their occupations (Collie et al., 2012; Skaalvik and Skaalvik, 2011; Yin et al., 2019).

Aside from leaders and colleagues, students play an irreplaceable role in school counselors’ well-being, as they provide the most direct feedback on counselors’ work and efforts. Perceiving student support signifies that school counselors and their work are valued, respected, and appreciated by students, which is usually central to counselors’ professional identity and goals (Butler, 2012; van der Want et al., 2015). The insufficiency of this support can lead to higher stress and reduced well-being in the occupation (Aldrup et al., 2018; Jo, 2014). Unfortunately, despite its significance, student support has often been neglected in studies of teachers’ and school counselors’ well-being (Aldrup et al., 2018).

Family support is also crucial, as it creates a mental refuge for school counselors to escape from occupational burdens and stress. Prior studies have shown that family support can boost school counselors’ positive psychological capital (e.g., optimism, self-efficacy, and resilience), thereby improving their job engagement, satisfaction and overall well-being (Chan et al., 2020; Huang et al., 2021; Kwok et al., 2015). Moreover, support from family members also alleviates the housework burden, especially for female counselors. As most Chinese school counselors are female and are usually expected to shoulder more household responsibilities, family support can help them better achieve work-family balance (Judge and Livingston, 2008; Xiang et al., 2023).

### 1.5 Basic psychological needs in occupation as significant mediators

In addition to situational and inter-individual supports, intra-individual resources also play a crucial part in protecting occupational well-being (Salmela-Aro et al., 2016; Salmela-Aro et al., 2022). Although numerous key personal resources affect occupational well-being (e.g., social–emotional skills, stress resilience), our study particularly focused on basic psychological needs (i.e., autonomy, competence, and relatedness). Exploring these needs can help us understand the dynamics between external supports and internal well-being for school counselors. In other words, these needs might serve as key mediators between situational or inter-individual supports and occupational well-being.

On the one hand, the Self-determination Theory proposes that individuals can only achieve a state of well-being when their basic psychological needs are satisfied (Deci and Ryan, 2000). Autonomy refers to the sense of freedom in individuals’ actions, while competence and relatedness refer to confidence in one’s abilities and feelings of connection with significant others, respectively (Deci and Ryan, 2000; Huang et al., 2024). These needs effectively enhance occupational positive affect, self-actualization, engagement, and satisfaction (Abós et al., 2018; Skaalvik and Skaalvik, 2014). Specifically, strong autonomy improves school counselors’ sense of ownership in their work, making them more motivated and self-initiated, which results in fewer occupational mental health problems (Neufeld and Malin, 2020; Stiglbauer and Kovacs, 2018). Similarly, school counselors with a strong sense of competence are likely to be more confident and less likely to experience burnout and negative affect (Klaeijsen et al., 2018; Salmela-Aro and Upadyaya, 2014). In addition, relatedness support is central to school counselors’ emotional and social adaptation, which is also essential for occupational well-being (Klassen et al., 2012; Zimmermann et al., 2018).

On the other hand, such basic psychological needs are largely influenced by situational and inter-individual supports in schools (Bakker and Demerouti, 2007). Situational supports create a supportive environment to nurture basic psychological needs (Ni et al., 2023), while social supports provide school counselors with feedback, guidance, and emotional support, which also help fulfill these needs (Bakker and Demerouti, 2017). Given these previous findings, it is expected that occupational autonomy, competence, and relatedness act as key mediators between multiple supporting factors and school counselors’ occupational well-being (Wang et al., 2022; Xiang et al., 2023). However, to date, what remains largely unexplored is which need satisfaction is more crucial to school counselors’ occupational well-being and which supporting factors can better satisfy these needs in the workplace.

### 1.6 The present study

**Figure 1:**
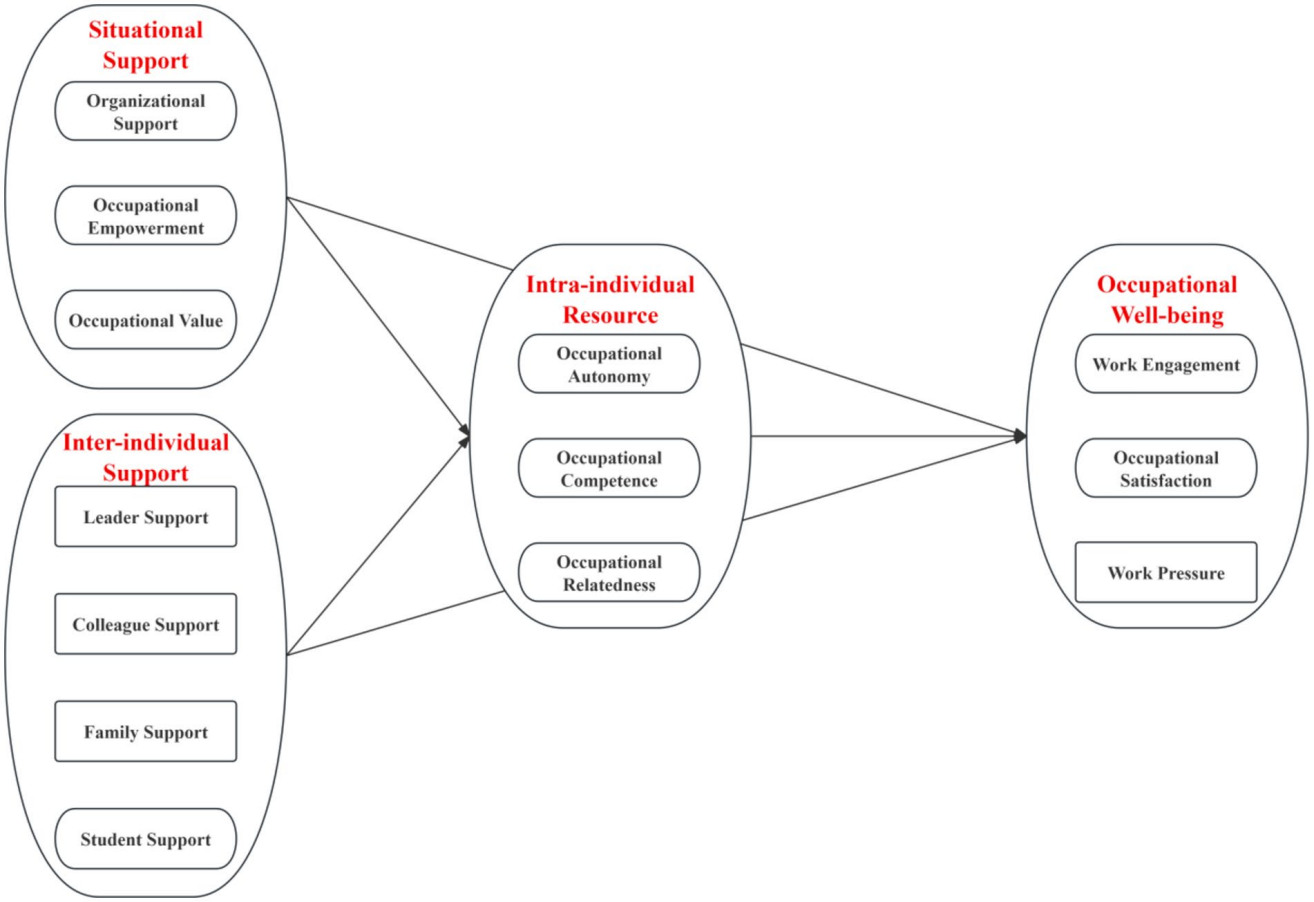
Theoretical framework.

In summary, although prior studies have investigated a wide scope of supporting factors for school counselors’ occupational well-being, few of them have attempted to identify the most effective strategies, limiting the practical efficacy of well-being facilitation. Moreover, part-time school counselors face more significant risks to their occupational well-being yet receive inadequate attention in existing literature. To address these research gaps, the present study aimed to comprehensively explore the associations among situational supports, inter-individual supports, basic psychological needs (i.e., as intra-individual resources), and school counselors’ occupational well-being. Furthermore, we explored the differences between full-time and part-time counselors. Based on the Demand-resource Model, we conducted the theoretical framework with the following research questions (see Figure 1):

1. Which supporting factors are robust and unanimous facilitators of all school counselors’ basic psychological needs and occupational well-being?
2. Which supporting factors have targeted benefits for part-time school counselors’ basic psychological needs and occupational well-being?
3. Which basic psychological needs play more prominent roles in school counselors’ occupational well-being, both in general and across different cohorts?

## 2 Method

### 2.1 Participants

The sample in this study comes from regional-representative datasets in Beijing, China. In total, 1,443 school counselors from primary schools to high schools were surveyed, including 482 full-time counselors and 961 part-time counselors (i.e., those also bearing teaching responsibilities). Among these participants, 459 (93.3%) full-time counselors and 876 (91.2%) part-time counselors are female.

Participation was fully voluntary, and informed consent forms were collected from both adolescents as well as their guardians and school teachers. The study was granted ethical permission by the research institute.

### 2.2 Measurement

All variables were reported by counselors themselves with good reliability and validity (Cronbach’s *α* > 0.75; in Confirmation Factor Analysis, CFI = 0.93, TLI = 0.92, RMSEA = 0.05, SRMR = 0.06; see factor loadings in Appendix).

*Organizational Support* was measured using a revised version of Perceived Organizational Support consisting of 11 items (Eisenberger et al., 1986; Liu et al., 2015), and demonstrated good reliability with Cronbach’s *α* = 0.96. An example item is: “Help is available from the organization when I have a problem at work.”

*Occupational Value* was assessed using the Chinese School Teachers’ Professional Identity Scale with four items (i.e., example item: I believe that school counselor is important to promote students’ holistic development; Wei et al., 2013). Good reliability was observed with Cronbach’s *α* = 0.94.

*Occupational Empowerment* was measured using the revised Psychological Empowerment Scale (Li et al., 2006; Spreitzer, 1995) with three items (i.e., example item is “My impact on what happens in my department is large”). Results showed good reliability (i.e., Cronbach’s *α* = 0.87).

*Inter-individual Support* includes support from school leaders, colleagues, students and family members. *Leader, Colleague and Family Supports* were reported by single items of “School leaders value my work in mental health counseling,” “Other school teachers support your work in mental health counseling” and “My family members support my work in mental health counseling,” respectively. *Student support* was measured using two items with good reliability (Cronbach’s *α* = 0.83): (1) “Students like the activities related to mental health,” and (2) “Compared with other teachers, students like me (as a school counselor) more.”

*Basic Psychological Needs* include autonomy, competence and relatedness needs.

Autonomy and competence were measured by the revised Psychological Empowerment Scale (Li et al., 2006; Spreitzer, 1995) with three and two items, respectively. The example items are “I have significant autonomy in determining how I do my job” and “I am confident about my ability to do my job in mental health counseling.” Relatedness was assessed using three items Chinese School Teachers’ Professional Identity Scale, with the example items of “I care about how others perceive my occupation as a school counselor.” Good reliability was observed in all three variables (i.e., Cronbach’s *α* = 0.84, 0.89, and 0.83).

*Work Engagement* was assessed using the Utrecht Work Engagement scale (Schaufeli and Bakker, 2003), which consists of eight items withgood reliability (Cronbach’s *α* = 0.95). An example item is “I feel strong and vigorous when I’m studying or going to work.” The Demands-resources Model conceptualizes engagement (in contrast to burnout) as a positive aspect of school-related well-being and psychological functioning (Salmela-Aro et al., 2022).

*Work Satisfaction* was measured using three items from the Job Satisfaction Scale (Schaffer, 1953) (Cronbach’s *α* = 0.79). Example item includes: “How much are you satisfied with the content of your job?”

*Work Pressure* was measured using a single item: “I feel stressful for my work in mental health counseling.”

*Covariants* included demographic variables of gender (0 = male, 1 = female), location, years of teaching experience, and education background.

### 2.3 Analysis

As the various independent variables and comparatively strong inter-correlation effects might contribute to the multicollinearity-related analytical bias, the authors adopted dominance analysis (DA) to ensure the reliability of the results, using Stata 17.0. Dominance analysis is a relative-importance determination method that estimates the general dominance weight (or Standardized Dominance Statistics, SDS) of independent variables and provides rank of importance. The dominance weight of variables is analyzed in a comparatively independent situation, and thus, this method can better address the bias due to multicollinearity and co-explained variations, compared with linear regression and structural equation models (Luchman, 2021). This survey was conducted by an online questionnaire without missing data since all items are compensatory to fulfill. The data and analytical methods (e.g., codes) are available for replication by contacting the research institute.

## 3 Results

### 3.1 Demographic information, descriptive analysis and correlation

First, the demographic information was introduced (see Table 1), and descriptive analysis and correlation were conducted (see Tables 2, 3). The results revealed that in both full-time and part-time school counselor cohorts, most of the independent variables and dependent variables showed moderate to strong correlations (*r* = 0.11–0.85), implying potential multicollinearity. As such, using dominance analysis was more advisable than linear regression and structural equation modeling to reduce statistical bias (Table 4).

**Table 1:** Demographic information of school counselors.

Variable	Full-time (*N* = 482)	Part-time (*N* = 961)
Gender	Male = 23; Female = 459	Male = 85; Female = 876
Teaching year	≤5 years = 164; 6–10 years = 131; 11–15 years = 105; 16–20 years = 61; ≥21 years = 21	≤5 years = 477; 6–10 years = 236; 11–15 years = 147; 16–20 years = 56; ≥21 years = 45
Education background	college or lower = 2; bachelor = 251; master or higher = 229	college or lower = 26; bachelor = 818; master or higher = 117
School type	primary school = 119; lower secondary school = 86; high school = 30; middle & high school = 127; school for 9- or 12-years education = 120	primary school = 651; lower secondary school = 140; high school = 12; middle & high school = 37; school for 9- or 12-years education = 121
School location	rural area = 45; small city = 51; suburbs of megalopolis = 70; center of megalopolis = 316	rural area = 323; small city = 81; suburbs of megalopolis = 151; center of megalopolis = 406

**Table 2:** Descriptive analysis and correlation of full-time school counselors.

	1	2	3	4	5	6	7	8	9	10	11	12	13	14	15	16	17	18
1. Gender																		
2. Location	−0.08																	
3. Teaching year	0.00	−0.01																
4. Education background	0.07	−0.21^***^	−0.20^***^															
5. Training support	−0.07	0.00	0.00	−0.14^***^														
6. Family support	−0.02	−0.01	−0.05	−0.05	0.25^***^													
7. Leader support	−0.08	−0.08	−0.08	−0.05	0.37^***^	0.36^***^												
8. Colleague support	−0.04	−0.05	−0.02	−0.11^*^	0.36^***^	0.41^***^	0.61^***^											
9. Student support	−0.03	0.01	0.01	−0.14^**^	0.19^***^	0.38^***^	0.29^***^	0.32^***^										
10. Organizational support	−0.07	−0.09	−0.13^**^	−0.09	0.40^***^	0.31^***^	0.70^***^	0.58^***^	0.37^***^									
11. Occupational empowerment	−0.06	−0.131^**^	−0.07	0.00	0.32^***^	0.28^***^	0.54^***^	0.48^***^	0.41^***^	0.78^***^								
12. Occupational value	0.03	−0.08	−0.07	−0.01	0.15^***^	0.23^***^	0.11^*^	0.12^***^	0.43^***^	0.18^***^	0.25^***^							
13. Work autonomy	−0.02	−0.06	−0.08	−0.04	0.32^***^	0.35^***^	0.51^***^	0.49^***^	0.45^***^	0.78^***^	0.79^***^	0.32^***^						
14. Work competence	0.06	−0.15^**^	0.04	0.11^*^	0.08	0.25^***^	0.16^***^	0.22^***^	0.40^***^	0.25^***^	0.50^***^	0.38^***^	0.45^***^					
15. Work relatedness	−0.11^*^	−0.04	−0.18^***^	0.01	0.16^***^	0.08	0.13^**^	0.07	0.27^***^	0.27^***^	0.32^***^	0.30^***^	0.27^***^	0.23^***^				
16. Occupational satisfaction	−0.09	−0.05	−0.08	−0.09^*^	0.35^***^	0.25^***^	0.61^***^	0.51^***^	0.29^***^	0.84^***^	0.75^***^	0.06	0.66^***^	0.20^***^	0.26^***^			
17. Occupational engagement	−0.04	−0.06	−0.08	−0.04	0.24^***^	0.42^***^	0.32^***^	0.30^***^	0.57^***^	0.49^***^	0.54^***^	0.46^***^	0.56^***^	0.53^***^	0.31^***^	0.44^***^		
18. Work pressure	−0.08	−0.05	0.05	0.06	−0.05	−0.12^***^	−0.05	−0.12^***^	−0.01	−0.14^***^	−0.15^**^	0.06	−0.13^**^	−0.09	0.02	−0.18^***^	−0.15^***^	
M	1.95	1.64	2.26	2.48	3.98	4.46	3.89	3.95	0.29	−0.07	0.06	0.20	0.15	0.48	0.07	−0.14	0.30	3.64
SD	0.21	1.00	1.18	0.52	0.92	0.61	0.91	0.73	0.70	0.91	0.88	0.81	0.79	0.65	0.95	0.90	0.80	0.99

^*^p < 0.05; ^**^p < 0.01; ^***^p < 0.001.

**Table 3:** Descriptive analysis and correlation of part-time school counselors.

	1	2	3	4	5	6	7	8	9	10	11	12	13	14	15	16	17	18
1. Gender																		
2. Location	−0.12^***^																	
3. Teaching year	−0.01	0.00																
4. Education background	0.10^**^	−0.14^***^	−0.11^**^															
5. Training support	0.05	−0.10^**^	0.01	0.06														
6. Family support	0.04	−0.09^**^	0.07^*^	0.04	0.43^***^													
7. Leader support	0.02	−0.15^***^	−0.06	−0.03	0.40^***^	0.44^***^												
8. Colleague support	−0.02	−0.11^**^	0.00	−0.01	0.39^***^	0.55^***^	0.72^***^											
9. Student support	0.08^**^	−0.10^**^	0.14^***^	0.00	0.47^***^	0.62^***^	0.40^***^	0.49^***^										
10. Organizational support	0.01	−0.11^**^	−0.05	0.00	0.43^***^	0.48^***^	0.74^***^	0.65^***^	0.50^***^									
11. Occupational empowerment	0.00	−0.15^***^	0.02	0.04	0.37^***^	0.47^***^	0.59^***^	0.54^***^	0.51^***^	0.80^***^								
12. Occupational value	0.08^*^	−0.03	0.06	−0.05	0.30^***^	0.43^***^	0.26^***^	0.33^***^	0.54^***^	0.37^***^	0.39^***^							
13. Work autonomy	0.05	−0.10^**^	0.02	0.03	0.42^***^	0.52^***^	0.58^***^	0.56^***^	0.60^***^	0.81^***^	0.84^***^	0.50^***^						
14. Work competence	0.01	−0.17^***^	0.17^***^	0.13^***^	0.28^***^	0.47^***^	0.26^***^	0.32^***^	0.54^***^	0.40^***^	0.59^***^	0.29^***^	0.53^***^					
15. Work relatedness	−0.01	0.00	0.00	0.06	0.20^***^	0.29^***^	0.23^***^	0.26^***^	0.36^***^	0.35^***^	0.41^***^	0.35^***^	0.38^***^	0.32^***^				
16. Occupational satisfaction	0.01	−0.08^*^	−0.06	0.01	0.36^***^	0.41^***^	0.63^***^	0.55^***^	0.41^***^	0.85^***^	0.76^***^	0.25^***^	0.68^***^	0.38^***^	0.33^***^			
17. Occupational engagement	0.01	−0.05	0.06	0.06	0.48^***^	0.66^***^	0.38^***^	0.45^***^	0.74^***^	0.58^***^	0.59^***^	0.51^***^	0.65^***^	0.62^***^	0.40^***^	0.52^***^		
18. Work pressure	0.03	0.04	0.03	−0.04	−0.04	−0.10^**^	−0.04	−0.06	−0.02	−0.13^***^	−0.13^***^	0.02	−0.10^***^	−0.14^***^	0.00	−0.19^***^	−0.13^***^	
M	1.91	2.33	1.91	2.09	3.93	4.10	3.92	3.96	−0.15	0.03	−0.03	−0.10	−0.08	−0.24	−0.04	0.07	−0.15	3.66
SD	0.28	1.32	1.14	0.37	1.00	0.84	0.99	0.84	0.99	1.02	0.98	1.03	1.01	1.00	0.95	0.95	1.03	0.95

^*^p < 0.05; ^**^p < 0.01; ^***^p < 0.001.

**Table 4:** Relative importance of supporting factors for basic psychological needs.

	Work autonomy				Work competence				Work relatedness			
	Full-time		Part-time		Full-time		Part-time		Full-time		Part-time	
	SDS	Rank	SDS	Rank	SDS	Rank	SDS	Rank	SDS	Rank	SDS	Rank
Gender	0.1%	11	0.2%	10	0.9%	11	0.1%	12	4.5%	7	0.3%	11
Location	0.2%	10	0.2%	9	2.6%	8	2.3%	11	0.2%	12	1.1%	10
Teaching year	0.2%	9	0.1%	11	1.3%	10	3.7%	5	**11.3%**	**5**	0.2%	12
Education background	0.1%	12	0.1%	12	3.2%	6	2.8%	9	0.3%	11	1.8%	9
Training support	2.8%	8	3.7%	8	0.8%	12	2.8%	10	4.6%	6	2.8%	8
Organizational support	**31.6%**	**2**	**25.5%**	**2**	7.8%	4	8.5%	4	**12.8%**	**4**	**13.6%**	**4**
Occupational value	4.1%	6	7.6%	5	**16.8%**	**2**	3.3%	7	**24.2%**	**1**	**20.1%**	**2**
Occupational empowerment	**34.8%**	**1**	**31.9%**	**1**	**41.5%**	**1**	**36.3%**	**1**	**20.9%**	**2**	**28.3%**	**1**
Leader support	8.4%	3	8.2%	4	1.9%	9	2.9%	8	2.6%	9	4.2%	7
Colleague support	7.3%	4	6.9%	6	2.9%	7	3.3%	6	3.3%	8	5.0%	6
Family support	3.5%	7	5.8%	7	4.7%	5	13.0%	3	1.2%	10	7.0%	5
Student support	6.9%	5	**10.0%**	**3**	**15.7%**	**3**	**20.9%**	**2**	**14.2%**	**3**	**15.8%**	**3**
Total *R*^2^	72.4%		79.7%		42.4%		50.3%		21.4%		23.1%	

SDS ≥ 10% are highlighted by bold.

### 3.2 Relative importance of supporting factors for basic psychological needs and occupational well-being

To identify the most effective facilitators for basic psychological needs and occupational well-being, dominance analysis was adopted to compare the relative importance of different supporting factors. The factors with strong standardized dominance statistics (i.e., SDS >10%) were highlighted. After controlling for gender, location, years of teaching experience, and educational background, the results in Table 5 indicated that occupational empowerment was the most robust and unanimously beneficial factor for all psychological needs in both full-time and part-time counselors (SDS = 34.8 and 31.9% for autonomy, 41.5 and 36.3% for competence, and 12.8 and 13.6% for relatedness in full-time and part-time counselors, respectively). Student support was also unanimously constructive (SDS = 10.0% for part-time counselors’ autonomy, and 15.7 and 20.9% for competence, 14.2 and 15.8% for relatedness in full-time and part-time counselors), except for the work autonomy of full-time school counselors (SDS = 6.9%). This support was more effective for part-time school counselors. Furthermore, the study found that organizational support was crucial to work autonomy (SDS = 31.6 and 25.5%)^1^ and relatedness (SDS = 12.8 and 13.6%) for both cohorts. Occupational value was critical for the work competence of full-time school counselors (SDS = 16.8%), as well as for work relatedness for both cohorts (SDS = 24.2 and 20.1%). Additionally, family support was only effective in the competence of part-time counselors (SDS = 13.0%).

**Table 5:** Relative importance of supporting factors and basic psychological needs for occupational well-being indicators.

	Work engagement				Occupational satisfaction				Work pressure			
	Full-time		Part-time		Full-time		Part-time		Full-time		Part-time	
	SDS	Rank	SDS	Rank	SDS	Rank	SDS	Rank	SDS	Rank	SDS	Rank
Gender	0.1%	15	0.1%	15	0.2%	13	0.0%	14	9.3%	5	1.9%	11
Location	0.2%	14	0.4%	12	0.1%	15	0.2%	13	4.0%	12	1.2%	14
Teaching year	0.4%	12	0.2%	13	0.2%	14	0.3%	12	3.7%	13	2.2%	10
Education background	0.2%	13	0.2%	14	0.3%	12	0.0%	15	4.1%	11	1.5%	13
Training support	1.4%	11	6.0%	8	2.8%	6	2.3%	9	0.6%	15	0.6%	15
Organizational support	9.7%	6	8.3%	5	34.1%	1	**34.7%**	**1**	**11.3%**	**2**	**16.6%**	**2**
Occupational value	**12.1%**	**3**	7.1%	7	1.5%	10	1.5%	11	9.7%	4	6.3%	7
Occupational empowerment	**10.8%**	**5**	7.4%	6	**22.5%**	**2**	**20.4%**	**2**	**11.1%**	**3**	**10.6%**	**4**
Leader support	2.8%	9	2.8%	11	**11.3%**	**4**	**10.3%**	**4**	7.8%	6	7.0%	6
Colleague support	2.4%	10	3.6%	10	6.9%	5	6.8%	5	6.9%	7	1.6%	12
Family support	9.1%	7	**15.1%**	**2**	1.2%	11	2.8%	8	**12.6%**	**1**	**11.3%**	**3**
Student support	**18.2%**	**1**	**21.3%**	**1**	1.7%	8	2.8%	7	4.4%	10	7.0%	5
Work autonomy	**11.9%**	**4**	**10.0%**	**4**	**13.6%**	**3**	**12.7%**	**3**	6.0%	8	5.6%	8
Work competence	**17.3%**	**2**	**13.7%**	**3**	1.6%	9	2.9%	6	5.7%	9	**22.1%**	**1**
Work relatedness	3.5%	8	3.8%	9	2.1%	7	2.2%	10	2.6%	14	4.7%	9
Total *R*^2^	56.3%		71.6%		75.6%		75.9%		8.3%		5.7%	

SDS ≥ 10% are highlighted by bold.

Then, this study included both supporting factors and basic psychological needs as independent variables to examine their contributions to occupational well-being. Regarding situational support, organizational support (SDS = 34.1 and 34.7% for occupational satisfaction, 11.3 and 16.6% for work pressure, but SDS scores were lower 10% in work engagement) and occupational empowerment (SDS = 10.8% for full-time counselors’ occupational satisfaction, 22.5 and 20.4% for occupational satisfaction, and 11.1 and 10.6% for work pressure) were comparatively unanimous facilitators of multiple well-being indicators for both full-time and part-time counselors, whereas occupational value only had a strong effect on full-time counselors’ work engagement (SDS = 12.1%). For inter-individual supports, we found that family support was important for work pressure (SDS = 12.6 and 11.3%) and part-time counselors’ work engagement (SDS = 15.1%), while student support was more effective for work engagement (SDS = 18.2 and 21.3%) and occupational satisfaction (SDS = 11.3 and 10.3%).

In terms of basic psychological needs, the study observed that work autonomy was crucial to work engagement (SDS = 11.9 and 10.0%) and occupational satisfaction (SDS = 13.6 and 12.7%) for both cohorts, and work competence was critical to part-time school counselors’ work pressure (SDS = 22.1%), as well as to work engagement for both full-time and part-time councilors (SDS = 17.3 and 13.7%). Therefore, factors satisfying these two needs could further facilitate these occupational well-being indicators. In contrast, the need for relatedness was less effective for all indicators.

## 4 Discussion

To effectively improve students’ well-being during school days, it is essential to first protect school counselors’ occupational well-being. Aiming to identify the most important well-being facilitators, the present study comprehensively examined the effects of situational, inter-individual, and intra-individual resources, as well as basic psychological needs, on full-time and part-time school counselors’ occupational well-being. In summary, the study found out that organizational support and occupational empowerment were unanimously robust facilitators for most psychological needs and well-being indicators, followed by student support, which particularly benefited part-time counselors. It also discovered that autonomy and competence support were potential mediators in transferring the benefits of supporting resources to occupational well-being. These findings extend the existing literature by highlighting the most effective occupational well-being facilitators both in general and specifically for part-time counselors, who often experience poorer occupational well-being, and thus imply targeted supporting strategies for their flourishing in their careers.

### 4.1 Organizational support and occupational empowerment as universally essential situational supports

This study first found that organizational support and occupational empowerment were universally prominent facilitators for both full-time and part-time school counselors’ well-being across multiple indicators, whereas occupational value only enhanced a few indicators. Although these three types of situational supports can stimulate school counselors’ motivation, engagement, and satisfaction in their occupation, their distinct functioning dynamics contribute to their varying effectiveness.

Organizational support and occupational empowerment provide immediate resources and support, enabling school counselors to access requisite development opportunities and appraisal guidance, while allowing them to have a say in deciding their work schedules and counseling strategies (Chen et al., 2022; Skår, 2010; Van Bogaert et al., 2016). These substantive supports and empowerment directly influence school counselors’ working conditions, thereby effectively promoting their occupational well-being (Lauermann and König, 2016; Skår, 2010; Van Bogaert et al., 2016).

In contrast, occupational value can only affect counselors’ occupational well-being through its indirect impacts on their beliefs and motivation. As the perceived significance and meaningfulness of occupation, occupational value is a macro-perspective on school counseling occupation, with less immediate linkage to counselors’ daily work (Fute et al., 2022; Jin and Rounds, 2012). Consequently, it is less sensitive to fluctuations in daily occupational well-being. However, a strong perceived occupational value is also related to greater emphasis on and richer support from schools (Balsamo et al., 2013; Sortheix et al., 2015; Sortheix et al., 2013). This explains its comparatively robust associations with work competence, relatedness, and engagement.

### 4.2 Students as the vital supporters, especially for part-time school counselors

Results also indicated that student played the most critical roles in satisfying school counselors’ basic psychological needs and benefiting their occupational well-being, especially for those working part-time. This finding is meaningful since their valuable support has been largely overlooked in the prior studies (Aldrup et al., 2018). Specifically, student support particularly fosters school counselors’ occupational competence, relatedness, and engagement. Students usually provide support through interaction and first fulfill the needs for relatedness. Furthermore, when school counselors feel their work is valued and appreciated by recipients (i.e., students), they are more inclined to generate a sense of competence, and to be more motivated to engage in their work (Butler, 2012; van der Want et al., 2015). However, as students can hardly impact school counselors’ work conditions and burdens, their support is less influential on counselors’ occupational autonomy, satisfaction and pressure.

Nonetheless, why is student support more targeted to benefit part-time counselors? Due to the lack of research comparing the effectiveness of support in different types of school occupations, we can only speculate. On the one hand, part-time school counselors often bear teaching tasks, thereby giving them more opportunities to interact with and be affected by students. On the other hand, many of them may be less professional in mental counseling and may fear doubts or criticism regarding their capabilities. In this situation, appreciation and recognition from students (i.e., as consultees) become more valuable and influential for their confidence and self-efficacy at work, thus impacting their well-being more constructively.

Besides student support, we found that family support was beneficial to reducing work pressure and enhancing part-time counselors’ competence and engagement. This result is understandable, since family members can buffer the burden of housework for school counselors, which helps them achieve a work-life balance and reduce related pressure (Judge and Livingston, 2008; Xiang et al., 2023). This support is more necessary for part-time counselors, as they usually carry heavier school workloads.

In addition, the results revealed that the leader and colleague support acted less effectively to facilitate school counselors’ occupational well-being. One explanation is that receiving support from colleagues or leaders may be interpreted as a sign of incompetence and this fear may yield diminishing returns for school counselors’ occupational well-being (Shin et al., 2020; Tews et al., 2013). Moreover, these supports may also contribute to mental burdens for school counselors, as they usually feel obligated to reciprocate (Poortvliet et al., 2009; Shin et al., 2020). These side effects may counteract the benefits of leader and colleague support, making them less constructive for school counselors’ occupational well-being.

### 4.3 Autonomy and competence as more central needs to occupational well-being

While all three needs were influenced by situational and inter-individual supports, only the satisfaction of autonomy and competence needs can effectively translate these supports into school counselors’ occupational well-being. This result partly aligns with the early assumptions of Self-determination Theory, in which autonomy and competence are more central needs for individuals than relatedness is (Deci and Ryan, 2000; Kashdan and Fincham, 2004).

On the one hand, the crucial roles of autonomy and competence are understandable. High autonomy allows school counselors to flexibly adopt individualized strategies for different students and arrange their workloads and schedules, which directly improves their work engagement and satisfaction (Neufeld and Malin, 2020; Stiglbauer and Kovacs, 2018). Meanwhile, strong perceived competence boosts school counselors’ confidence in their work, so they are more engaged and can better handle work pressure (Klaeijsen et al., 2018; Salmela-Aro and Upadyaya, 2014). Competence is particularly essential to part-time counselors’ work well-being, who often lack sufficient training and feel less self-efficient about their work. Thus, satisfying their needs for competence can effectively prevent this well-being risk.

Nonetheless, why is relatedness a less central need to school counselors’ occupational well-being? A possible explanation is that relatedness is a more general need in life, whereas autonomy and competence can be occupation-specific needs that directly impact school counselors’ occupational well-being (Deci and Ryan, 2000; Kashdan and Fincham, 2004). In other words, despite the significance of relatedness (as a need), it could hardly affect the specific aspects of well-being immediately, especially in the school counselors’ occupation, which involves comparatively independent work (i.e., most of the time they only interact with students in one-on-one counseling). However, as few prior studies have compared the effectiveness of school counselors’ different basic psychological needs, this result and the related explanation need further examination.

### 4.4 Implications

The findings in this study have several implications for benefiting school counselors’ occupational well-being. First, given the universally constructive roles of organizational support and occupational empowerment, school managers should create a supportive organizational environment with clear communication channels, regular feedback sessions, and recognition programs to acknowledge the contributions of school counselors. They are also expected to involve school counselors in school decision-making processes, particularly on issues that directly affect their work.

Second, as students provide the most critical inter-individual support, especially for part-time counselors, it is essential to strengthen positive interactions between counselors and students, such as providing opportunities for school counselors to interact with students outside of formal counseling sessions and encouraging communication and feedback from students.

Finally, the study identified a greater priority to satisfy school counselors’ autonomy and competence needs, and thus, schools should grant counselors greater flexibility in their work, ensure they have the necessary tools and resources, and offer specialized training to enhance their occupational competence.

### 4.5 Limitations and future directions

There are some limitations in our study. First, since few previous studies have been conducted in this field, our study is only exploratory; it is necessary to re-examine and explore the results and their underlying dynamics in further studies. Second, the cross-sectional nature of our data prevents us from establishing causal relationships among situational and inter-individual supports, basic psychological needs, and occupational well-being. Future studies are expected to identify the most prominent well-being predictors through longitudinal surveys. Finally, to reduce respondent burden, some variables were measured by single items (i.e., leader, colleague, family supports, and work pressure), which might contribute to some bias in the results.

## 5 Conclusion

By comprehensively examining the associations among situational supports, inter-individual supports, basic psychological needs, and school counselors’ occupational well-being, this study identified the most effective situational supports (i.e., organizational support and occupational empowerment), vital supporters (i.e., students) and the most central psychological needs (i.e., autonomy and competence) in promoting their occupational well-being. The findings imply effective supporting strategies to improve school counselors’ work conditions and address well-being risks, helping guarantee their work efficacy in protecting children’s mental health.

## Data Availability

The raw data supporting the conclusions of this article will be made available by the authors, without undue reservation.

## Footnotes

^1^In this section, the left-hand score represents the SDS results for full-time counselors, while the right-hand score is the SDS results for part-time counselors.

## Appendix

**Table A1:** Factor loadings of latent variables.

Variable	Factor loadings	Item
Organizational support	0.82	The organization values my contribution to its well-being
	0.77	The organization cares about my general satisfaction at work
	0.87	Help is available from the organization when I have a problem in work
	0.85	Help is available from the organization when I have a problem in life
	0.64	The organization would understand a long absence due to my personal reasons
	0.88	The organization strongly considers my goals and values.
	0.88	The organization really cares about my well-being
	0.86	The organization is willing to extend itself in order to help me perform my job to the best of my ability
	0.82	The organization would grant a reasonable request for a change in my working conditions
	0.86	The organization takes pride in my accomplishments at work
	0.90	The organization wishes to give me the best possible job for which I am qualified
Leader support	/	School leaders value my work in mental health counseling
Colleague support	/	Other school teachers support your work in mental health counseling
Student support	0.87	Students like the activities related to mental health
	0.81	Compared with other teachers, students like me (as a school counselor) more
Family support	/	My family members support my work in mental health counseling
Training support	/	The school training for mental health counseling is helpful for my work
Occupational value	0.92	I believe that school counselor is important to promote students’ holistic development
	0.91	I believe that school counselor is important for school education
	0.92	I believe that school counselor is beneficial to students’ academic progress
	0.80	I believe that every school should have at least one school counselor
Occupational empowerment	0.83	My impact on what happens in my department is large
	0.81	I have a great deal of control over what happens in my department
	0.86	I have significant influence over what happens in my department
Autonomy	0.85	I have significant autonomy in determining how I do my job
	0.86	I can decide on my own how to go about doing my work
	0.70	I have considerable opportunity for independence and freedom in how I do my job
Competence	0.86	I am confident about my ability to do my job in mental health counseling
	0.95	I can professionally help student improve their mental health
Relatedness	0.71	I care about how others perceive my occupation as a school counselor
	0.75	I feel insulted when someone makes unwarranted accusations about the school counselor group
	0.94	I care about how others perceive the community of school counselors
Work engagement	0.85	When I’m doing my work as a school counselor, I feel bursting with energy.
	0.86	I feel strong and vigorous when I’m studying or going to work
	0.91	I am enthusiastic about my work
	0.90	I find my work full of meaning and purpose
	0.85	I am proud of my work
	0.68	I feel happy when I am working intensely
	0.86	When I am working, I forget everything else around me
	0.82	Time flies when I am working
Work satisfaction	0.81	How much are you satisfied with your salary?
	0.87	How much are you satisfied with the promotion opportunities in your organization?
	0.59	How much are you satisfied with the content of your job?
Work pressure	/	I feel stressful for my work in mental health counseling
